# Sex differences in a murine model of infective endocarditis

**DOI:** 10.1007/s00395-025-01127-8

**Published:** 2025-08-06

**Authors:** Benedikt Bartsch, Raúl Nicolas Jamin, Axel Schott, Muntadher Al Zaidi, Nikola Lübbering, Hannah Billig, Christian Kurts, Georg Nickenig, Marijo Parcina, Sebastian Zimmer, Christina Katharina Weisheit

**Affiliations:** 1 Heart Center Bonn, Department of Medicine II, University Hospital Bonn, Bonn, Germany; 2 Institute of Molecular Medicine and Experimental Immunology, University Bonn, Bonn, Germany; 3 Institute of Medical Microbiology, Immunology and Parasitology (IMMIP), University Hospital Bonn, Bonn, Germany; 4 Department of Anaesthesiology and Intensive Care Medicine, University Hospital Bonn, Bonn, Germany

**Keywords:** Infective endocarditis, Sex differences, Murine model, Immune response, Staphylococcus aureus, Valve inflammation

## Abstract

Infective endocarditis (IE) is a highly lethal disease with a notable male predominance, yet the biological basis for this sex disparity remains unclear. We established a murine IE model in C57BL6 mice in which aortic valve injury was induced via wire-injury and followed by intravenous injection of Staphylococcus aureus. Infection was confirmed by blood and valve cultures, and cardiac function was evaluated by echocardiography. Systemic cytokine levels were measured, and immune cell infiltration in valve tissue was assessed by flow cytometry and immunofluorescence. In the murine model, IE was induced in 77/85 animals. Male mice exhibited significantly higher bacterial loads in blood and valves, greater valve cusp enlargement, increased ventricular volumes, and more frequent aortic regurgitation. Both sexes showed strong neutrophilic responses, but males had markedly elevated systemic IL-1α, IL-1β, IL-6, and TNF-α levels. Females demonstrated earlier and more robust recruitment of CD68⁺ and CD206⁺ macrophages, as well as Ly6G⁺ neutrophils, to the injured valve, correlating with reduced bacterial vegetations. This murine model mirrors the clinical sex disparity in IE: males develop more severe disease and systemic inflammation, while females benefit from a rapid, localized immune response. These findings provide a platform for dissecting molecular drivers of sex-specific susceptibility in IE.

**Supplementary Information:**

The online version contains supplementary material available at 10.1007/s00395-025-01127-8.

## Background

Infective endocarditis (IE) is the fourth deadliest infectious disease worldwide, with rising mortality rates globally since the 1990s [8, 23]. Its main risk factors include prior IE infection, the presence of prosthetic valves, ventricular assist devices, or congenital heart disease [8]. Despite optimal medical treatment, the in-hospital mortality rate remains almost 30% [24].

The proportion of females among IE patients varies between studies and their geographic origins. There is a clear tendency toward men comprising the majority of the IE patient collective [32, 33]. However, recent data highlight an excessive in-hospital mortality rate for women in IE when adjusting for variables such as age or prosthetic valve disease [21, 22].

It remains unclear whether these gender-specific observations can be attributed to differences in healthcare response such as a later initiation of therapy due to less specific symptoms or to a more conservative surgical indication, or whether sex-specific differences in the immune response to bacterial infections—particularly in IE—must be considered [6, 28, 31].

In recent years, sex differences in the innate and adaptive immune response have become apparent. Females exhibit a faster and more robust immune response to bacterial infection in its initial stages [9, 14]. This is reflected in a significantly increased cytokine release in females following bacterial infection [9, 40]. Interestingly, this sex difference depends on the bacterial dose in both humans and experimental animals. In moderate infections, females show an initially enhanced systemic inflammatory response, whereas in the course of severe bacterial infections, an excessive cytokine storm is observed in males, which is associated with increased complications [11, 37, 40].

In bacterial infections in females, the cellular immune response shows an initially increased infiltration of immune cells at the sites of infection, particularly neutrophils and macrophages, along with an enhancement of the phagocytic function of these cells. This is associated with an increased pathogen clearance in females [4, 17, 30]. In males, a testosterone-dependent reduction in the ratio of anti-inflammatory M2 to pro-inflammatory M1 cells is observed [9, 14].

The underlying pathophysiology is complex and not yet well understood. Studies have shown that there are differences in the expression of various Toll-like receptors between macrophages and neutrophils of male and female subjects [10, 14]. Additionally, it has been demonstrated that female cells respond more strongly to both self and foreign antigen presentation, which, on one hand, promotes the occurrence of autoimmune phenomena but, on the other hand, facilitates rapid pathogen clearance in bacterial infections and can help prevent their spread [10, 39]. In vitro, a testosterone-mediated difference in cytokine expression is observed in isolated monocytes between the sexes. In male cells, there is an increased production of pro-inflammatory cytokines, such as TNF-alpha, following stimulation with LPS, while female cells show an enhanced response of anti-inflammatory cytokines, such as IL-10 [10]. Interestingly, the microbiome has been demonstrated to play an important role in immune system homeostasis and also shows sex specific differences [19, 35].

Sex differences in IE have not yet been analyzed in vitro or in vivo, despite the differing prevalence between sexes. We recently developed a novel IE model based on ultrasound-guided endothelial damage via wire injury that can consistently induce IE in mice [2]. Our aim in this study was to explore potential sex differences in IE induction.

## Methods

### Mice

Male and female C57BL/6-J (wild-type) mice, aged 10–14 weeks, were obtained from Janvier Labs, France. The animals were provided with ad libitum access to water and food. They were housed in a facility maintained at 22 °C with a 12 h light–dark cycle. All experimental procedures were conducted in compliance with the guidelines approved by the local governmental Animal Ethics Committee.

### Echocardiography

Mice were anesthetized with 1.5% isoflurane for echocardiographic assessment, with continuous monitoring of respiratory rate, electrocardiogram, and body temperature. Aortic valve function was evaluated using the suprasternal view to measure peak velocity, assess regurgitation, and detect bacterial vegetations. Standard echocardiographic parameters, including left ventricular ejection fraction, fractional shortening, and ventricular volumes, were obtained from parasternal long-axis views. Aortic regurgitation severity was classified as mild (jet immediate to the aortic valve), moderate (jet extending to the length of the left ventricular outflow tract), or severe (jet extending beyond the outflow tract).

### Wire injury

The wire injury (WI) procedure was conducted following a protocol previously established by our institution [25]. Briefly, the mice were anesthetized with an intraperitoneal injection of 150 mg/kg Ketamine and 16 mg/kg Xylazine. The right carotid artery was surgically accessed, and a straight guidewire with a shortened and soldered tip (Abbott HI-TORQUE 0.014) was introduced. Blood flow was temporarily occluded using two ligatures. Echocardiography was utilized to verify proper wire placement within the left ventricle through the trans-aortic valve. To induce endothelial damage, the wire was moved back and forth across the valve 50 times and rotated 100 times. Continuous echocardiographic monitoring was performed during this process to ensure there was no occurrence of aortic regurgitation. Following this, the wire was withdrawn and the artery was ligated.

### Bacterial challenge and cultivation

The methicillin-sensitive *Staphylococcus aureus* strain SA-LT 68/03C12Y7, isolated from human specimens, was cryopreserved at − 80 °C in a 20% glycerol solution. For the preparation of the bacterial challenge (BC) suspension, S. aureus was cultured in LB medium for 4 h at 37 °C with gentle agitation. The bacteria were then suspended in sterile 0.9% NaCl to achieve a concentration of 10^5^ CFU/100 μl. The bacterial concentration was confirmed by inoculating 10 μl of the suspension onto 5% blood agar plates (BD Biosciences). The BC was administered via intravenous injection of 0.1 ml of the bacterial suspension three days following wire injury (WI). After injection, blood cultures for *S. aureus* were collected from the animals and incubated on three different 5% blood agar plates, bacterial cultures were counted, and mean values were calculated based on the agar plates.

### Histological preparation

The mice were euthanized by cervical dislocation at 1, 3, or 7 days post-bacterial challenge. Hearts were excised using sterile instruments and flushed with sterile 0.9% saline solution. The hearts were then prepared for immunofluorescence analysis or Gram staining. Hearts were bisected through a sagittal incision along the left ventricular outflow tract under a light microscope at 10 × magnification. Residual blood clots were removed with sterile saline solution. Immune cell infiltration and macrophage presence in the aortic valve were visualized using CD68 and CD45 staining techniques as described by Niepmann et al. [25]. For Ly6G staining, aortic valve sections were fixed in acetone for 30 min and blocked with 10% normal goat serum (NGS). The primary antibody (anti-Ly6G, rat 1A8 anti-mouse, BD Biosciences, USA) was diluted to 1:200 and incubated overnight, followed by incubation with the secondary antibody (Cy3 AffiniPure Donkey anti-Rat IgG, Jackson ImmunoResearch Laboratories Inc) diluted to 1:500 for 90 min. For S. aureus staining, aortic valve sections were fixed in 0.1% Saponin for 30 min and blocked with 10% NGS. Nuclear counterstaining was performed using NucBlue™ (DAPI) (ThermoFisher Scientific, USA).

### Flow cytometry

To quantify immune responses, 50 µl of whole blood was collected from each mouse. Red blood cells were removed by filtration, and Fc receptors were blocked using Fc-Block (Pharmingen). Following washing steps, cells were stained with antibodies against Ly6G, B220, CD11b (BioLegend), CD3 (eBioscience), CD45, and NK1.1 (BioLegend). Flow cytometry analysis was conducted using a FACSCanto II (BD Bioscience, Franklin Lakes, USA). Data analysis was performed using FlowJo software (Tree Star, Ashland, USA).

### Cytokine expression

Immune responses in peripheral blood samples were quantified using a Luminex-ELISA kit (Mouse XL Cytokine Luminex^®^ Performance Premixed Kit) according to the manufacturer’s protocol. A 50 µl volume of murine plasma was used for each assay. The panel of analytes detected included IL-1α, IL-1β, IL-6, IL-10 and TNF-α.

### Statistical analysis

Data are expressed as mean ± standard error of the mean (SEM). Prior to statistical analysis, the data were assessed for assumptions of normality and homogeneity of variance between experimental groups. The number of experiments and mice per group are specified in the figure legends. For comparisons between two groups, Student’s *t* tests were employed. For multiple comparisons, one-way or two-way analyses of variance (ANOVA) followed by Tukey’s post-hoc test were used. Statistical analyses were performed using GraphPad Prism version 8 (GraphPad Software Inc., La Jolla, CA). The results are presented as mean unless otherwise indicated. *P* values < 0.05 were considered statistically significant. Based on registry data, an analysis of the sex distribution of all endocarditis patients treated at the University Hospital Bonn from 2013 to 2023 was conducted.

## Results

Based on an increased incidence of endocarditis in men, as previously described in the literature, we aimed to use our endocarditis mouse model to analyze to what extent, aside from disparities in lifestyle and risk exposure, sex-specific differences in vulnerability to bacterial IE exist.

### Bacterial burden in IE in mice

**Fig. 1:**
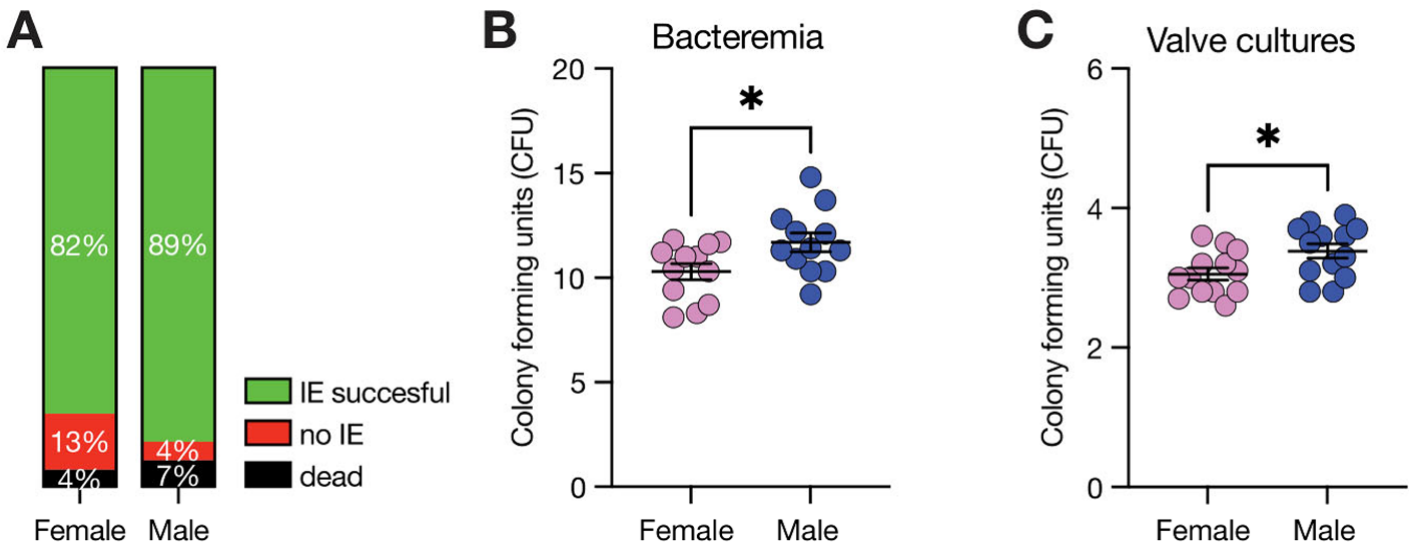
Gender Analysis of Infective Endocarditis. **A** Efficacy of IE induction in female and male mice. (B) Bacteremia in murine IE model was assessed 24 h after bacterial injection and was more prevalent in male than female mice, correspondingly valvular bacterial infiltration was increased in male mice as well (C). Data is presented as mean ± SEM. *P < 0.05. *CFU* colonies forming units, *IE* infective endocarditis

Ninety animals were included in this study. Five died following bacterial injection (two males, three females) and IE induction was successful in 37/43 female and 40/42 male mice (Fig. 1A). Bacteremia, a prerequisite for IE development, was assessed 24 h following bacterial challenge. The number of colony forming units (CFU) was higher in blood of male mice compared to females (female: 10.29 ± 1.34 CFU vs. male: 11.69 ± 1.55 CFU; *p* = 0.03; Fig. 1B). Similarly, bacterial colonization of the valves was more pronounced in male mice (female: 3.05 ± 0.32 CFU vs. male: 3.48 ± 0.38 CFU; *p* = 0.004; Fig. 1C).

### Aortic valve damage and myocardial changes

**Fig. 2:**
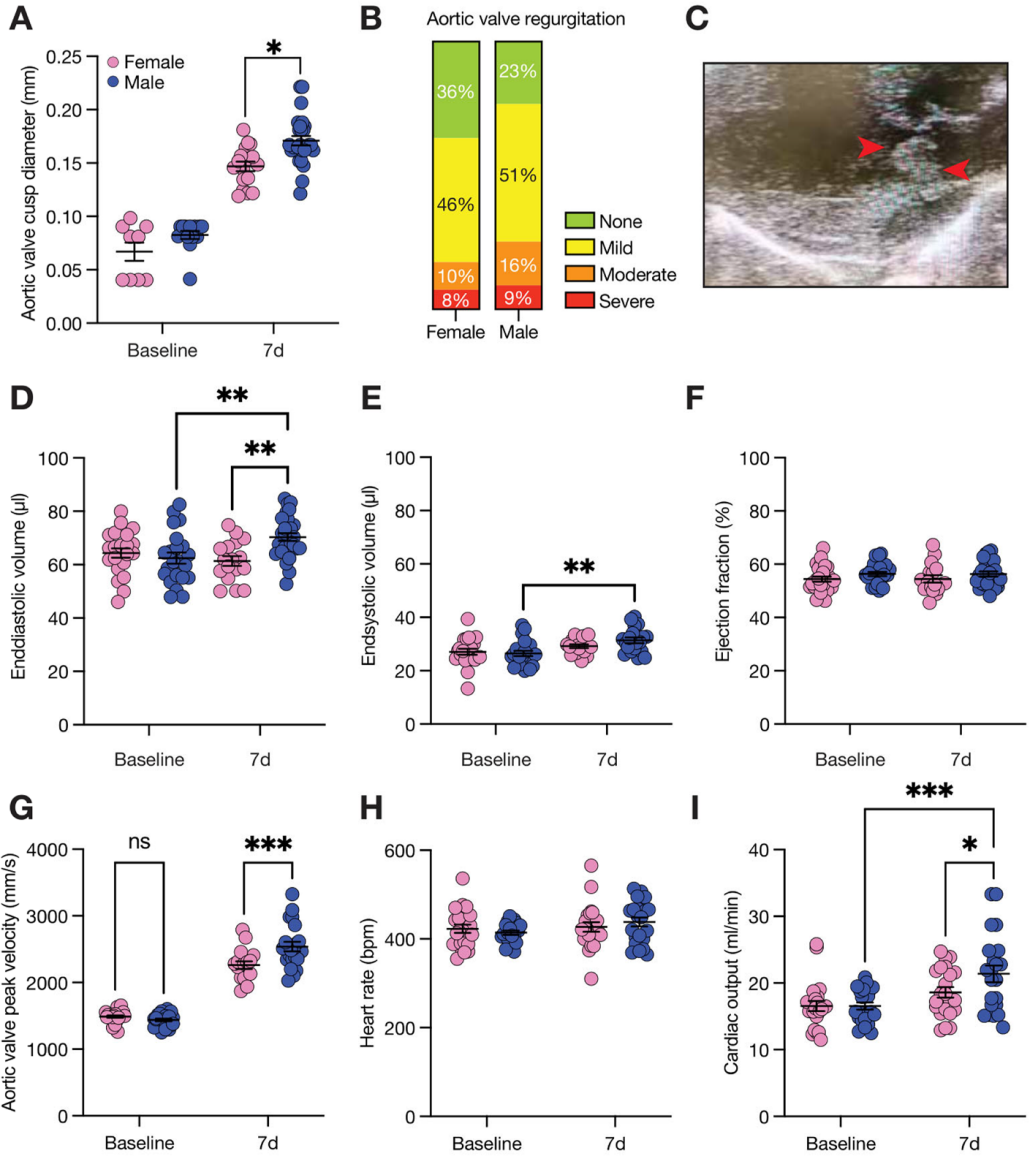
Valvular and myocardial changes in murine IE model. Transthoracic echocardiography was performed at baseline and day 7 after bacterial challenge. Aortic valve cusp diameter (**A**) and aortic valve regurgitation (**B**) are indirect parameters of IE severity. (C) Exemplary image of aortic valve vegetation (red arrows) in parasternal long axis view. To assess valvular and myocardial changes, ventricular volumes and left-ventricular ejection fraction, aortic peak velocity, heart rate and cardiac output (**D**–**I**) were measured. Data is presented as mean ± SEM, **P < 0.01; *P < 0.05

Echocardiography was performed before wire-injury and day 7 after bacterial challenge to quantify valvular and myocardial changes upon IE induction [2, 25]. Aortic cusp diameter and aortic valve regurgitation, serving as surrogate markers for bacterial vegetations and valvular injury, were assessed using parasternal long- and short-axis views (Fig. 2A, B). Figure 2C provides an exemplary image of pronounced valvular bacterial vegetations, underscoring the utility of transthoracic echocardiography in this model. Post-bacterial challenge, the aortic cusp diameter increased compared to baseline, with a more pronounced thickening in male mice (Fig. 2A). Aortic valve regurgitation was also more frequent and of greater severity in male animals (Fig. 2B). Among the five animals that died during this study, all exhibited severe aortic regurgitation. In line with these findings, male mice exhibited an increased end-diastolic and end-systolic volume (Fig. 2D, E), while ejection fraction (Fig. 2F) remained unchanged in both sexes. No differences between sexes were observed in heart rate (Fig. 2F). Aortic peak velocity increased after wire injury, as described by Niepmann et al., and was further elevated in males compared to females 7 days post-infection, indicating sex-specific differences in aortic stenosis progression (Fig. 2G). Because heart rate was similar in all animals (Fig. 2H), stroke volume (not shown) and cardiac output (Fig. 2I) increased after 7 days compared to baseline and were also higher than in males at 7d after bacterial challenge.

### Systemic cytokine response

Given the increased local and systemic bacterial load, alongside a higher prevalence of valvular and myocardial damage in male animals, we sought to investigate potential underlying immunological mechanisms. Specifically, we aimed to compare systemic and local immune responses between sexes to determine whether sex-specific differences in host immunity contribute to the observed disparity in bacterial burden.

To assess the systemic immune response, we first analyzed cellular immune profiles using flow cytometry from whole blood and cytokine levels from plasma samples collected immediately before sacrifice at 1, 3, and 7 days post-bacterial challenge. A pronounced neutrophilia was observed early after infection (day 1 and day 3), accompanied by an early decline in circulating B and T cells. However, no significant differences were detected between male and female animals in neutrophil, lymphocyte or monocyte counts.

**Fig. 3:**
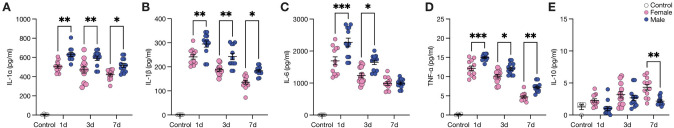
Plasma cytokine levels following IE induction. Cytokines were measured on day 1, day 3 and day 7 after bacterial challenge. The pro-inflammatory cytokines IL-1α (**A**), IL-1β (**B**), IL-6 (**C**), TNF-α (**D**) were increased in male mice compared to females after bacterial challenge. While circulating levels of the anti-inflammatory cytokine IL-10 (E) rose over time it was consistently lower in male mice. Data are presented as mean ± SEM. ***P < 0.001; **P < 0.01; *P < 0.05

Next, the systemic cytokine response was quantified using a murine LUMINEX XL assay. A sharp increase in circulating pro-inflammatory cytokines (IL-1α, IL-1β, IL-6, TNF-α) was detected in both sexes immediately following bacterial challenge and gradually declined over time (Fig. 3A–D). Notably, these pro-inflammatory cytokines were consistently higher in male mice compared to females, particularly at day 1 and day 3 post-infection (Fig. 3A–D). In contrast, the anti-inflammatory cytokine IL-10 did not differ between sexes on day 1 and day 3, but was significantly elevated in female mice day 7 post-infection (Fig. 3E).

### Valvular immune cell infiltration

**Fig. 4:**
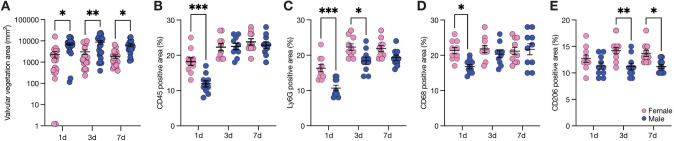
Immune cell infiltration of the aortic valve. Aortic valve cross sections were obtained immediately after sacrifice. Valvular vegetation area was assessed using Gram staining (A). Valvular immune cell infiltration was analyzed using immunofluorescence microscopy after CD45 (B), Ly6G (C), CD68 (D) and CD206 (E) staining day 1, day 3 and day 7 after bacterial challenge. Data is presented as mean ± SEM, and statistical significance was determined using unpaired/paired one-way ANOVA. ***P < 0.001; **P < 0.01; *P < 0.05. *BC* bacterial challenge, *WI* wire-injury

To examine whether temporal changes in systemic inflammation are reflected in bacterial colonization, we quantified valvular vegetations using Gram staining (Fig. 4A). Consistent with valve cultures (Fig. 1D), larger vegetations were observed in male compared to female animals on day 1 (Fig. 4A). This trend persisted over the duration of the observed time points, but the largest vegetations were measured on day 3 after bacterial challenge (Fig. 4A).

Valvular immune cell infiltration was assessed to investigate the potential relationships between bacterial load and inflammatory response. Interestingly, valves from female mice displayed a more pronounced infiltration with CD45^+^, Ly6G^+^ and CD68^+^ cells on day 1 following bacterial challenge (Fig. 4B–D). By day 3, valvular immune-cell infiltration was similar between the sexes, with only Ly6G^+^ cells being more prevalent in valves of female mice (Fig. 4C). Of note, a higher proportion of CD206^+^ macrophages was observed on day 3 and day 7 after bacterial challenge in aortic valves of female mice (Fig. 4E).

## Discussion

Epidemiological studies, including our own observations, have consistently demonstrated a pronounced male predominance in IE incidence. This study sought to determine if these disparities are also evident in a mouse model of IE and whether intrinsic differences in immune response contribute to male susceptibility to IE.

To investigate these disparities in IE, we utilized a recently developed IE model based on ultrasound-guided wire-injury induced endothelial damage followed by a single intravenous S. aureus bacterial challenge [2]. Even though the number of viable bacteria injected was identical in both male and female mice, bacteremia, as a necessary precursor for IE development, were more pronounced in male mice compared to females and the number of CFU isolated from valvular cultures was also greater in male samples. A higher bacteremia burden in male animals was previously described in several different bacterial murine models, including urinary tract infections, sepsis and tuberculosis [7, 20, 29].

One important distinction between the IE model used in this study compared to previously published models is the much lower initial bacterial challenge allowing for a pathophysiological differentiation between infiltrative IE and systemic sepsis [12, 15, 26, 27]. In fact, the comparatively high overall survival rate of 94% allows for a longer follow-up and also more closely simulates human disease.

Bacteremia, plasma cytokines, and valvular immune cell infiltration were more pronounced in male experimental animals. This is consistent with studies in both humans and mice that have shown a heightened and often dysregulated immune response promoting secondary tissue damage in males compared to females [11, 37, 40]. We do, however, find an increased valvular immune cell infiltration on day one after surgery in female mice. This very early immune response could effectively dampen IE severity in female mice resulting in a decreased systemic inflammation. While comparative data on sex-specific differences in immune cell responses during IE are scarce, studies with other bacterial infections have demonstrated similar findings with a faster and more effective immune cell infiltration in female experimental animals and improved bacterial clearance [4, 10, 13, 17, 30].

The sustained elevation of cytokine levels observed in our experiments cannot be attributed solely to bacterial challenge, as these levels persisted beyond seven days and coincided with a male-specific increase in valvular bacterial infiltration. This suggests that prolonged cytokine expression is more likely driven by the progression of severe IE rather than the transient bacterial stimulus.

Systemic cellular immune response revealed an early neutrophilia in both sexes with relatively reduced lymphocyte counts. However, pro-inflammatory cytokines in plasma samples were higher in male samples than females after bacterial challenge.

In valvular samples immune cell infiltration was earlier (day 1 and day 3 after BC) in females compared to males. Female mice showed an increase in CD206^+^ macrophage infiltration compared to males, day 1, day 3 and day 7 after bacterial challenge.

CD206 serves as a surface marker for the classification of various macrophage subtypes [5, 38]. It is predominantly expressed by M2 macrophages, which are associated with anti-inflammatory responses and tissue repair [38]. This receptor plays a pivotal role in the immune system by mediating the recognition and internalization of pathogens [5, 36]. Previous studies have linked an increased infiltration of CD206^+^ macrophages into tissues with elevated expression of anti-inflammatory cytokines such as IL-10 [3, 18]. Furthermore, a higher M2 macrophage ratio in females during localized bacterial infections has been reported in other animal models (Deny et al., 2022).

Our findings also demonstrate a time-dependent increase in IL-10 levels, which is more pronounced in female animals. Consistently, we observed greater infiltration of CD206^+^ cells in female animals. CD206^+^ cells have been widely associated with enhanced tissue repair processes and reduced chronic inflammation [34].

While more recent studies report increased in-hospital mortality for women with IE, we did not observe a biological correlate for this finding in our animal model. This could suggest that, in addition to biological factors, healthcare-related disparities may contribute to gender differences and should be considered in future investigation [21, 22].

The differences in aortic peak velocity also provide evidence for potential sex differences as a contributing factor in the development of aortic valve stenosis [1].

In summary, our study underscores differences in the immune response in an animal model of IE between the sexes. Translating these findings into the clinical context requires caution, given the additional influence of environmental and risk factors in patients. Also, the model can be used to further investigate molecular mechanisms differentiating gender aspects and to test potential therapeutics protocols.

Due to the study design, there are limitations. This is an animal study conducted on mice, and the immunological response to bacterial diseases differs between species (human and mouse). The induction of IE is achieved by intravenous administration of S. aureus, which is indeed the most common pathogen causing IE but by no means the only one [8]. Furthermore, our strain was isolated from patients; however, S. aureus strains can vary significantly in their tissue toxicity. Another possible limitation is the fundamental difference between murine and human immune systems [16]. Also, because overall mortality was very low in our model no conclusions may be drawn in this context. Future studies with higher bacterial loads and longer observation periods may clarify whether female mice show increased mortality after IE induction.

In this study, we demonstrate differences in the immune response to IE between male and female mice. Bacterial clearance occurs more effectively and rapidly in female animals, showing a less pronounced systemic inflammatory response. Additionally, there is a different infiltration of immune cells in the heart valve tissue, which may reduce the development aortic stenosis in female animals.

## Supplementary Information

Below is the link to the electronic supplementary material.

Supplementary file1 (DOCX 95 KB)

## Data Availability

Additional information required to reanalyze the data reported in this paper is available from the lead contact upon request.
