# Supplementary material for: Sex differences in a murine model of infective endocarditis
Source: Basic Res Cardiol. 2025 Aug 6;120(5):1027–35. doi: 10.1007/s00395-025-01127-8 (PMC12518371; doi:10.1007/s00395-025-01127-8)
Supplement: Supplementary file 1 — Supplementary file1 (DOCX 95 KB) [file 395_2025_1127_MOESM1_ESM.docx]

Sex differences in a murine model of Infective Endocarditis

^a^Benedikt Bartsch*, ^a^Raul Nicolas Jamin*, ^a^Axel Schott, ^a^Muntadher Al Zaidi, ^a^Nikola Lübbering, ^a^Hannah Billig, ^b^Christian Kurts, ^a^Georg Nickenig, ^c^Marijo Parcina ^a^Sebastian Zimmer*, ^d^Christina Katharina Weisheit*

^a^ Heart Center Bonn, Department of Medicine II, University Hospital Bonn, Bonn, Germany

^b^ Institute of Molecular Medicine and Experimental Immunology, University Bonn, Bonn, Germany

^c^ Institute of Medical Microbiology, Immunology and Parasitology (IMMIP), University Hospital Bonn, Bonn, Germany

^d^ Department of Anaesthesiology and Intensive Care Medicine, University Hospital Bonn, Bonn, Germany

Corresponding author: Benedikt Bartsch (benedikt.bartsch@ukbonn.de)


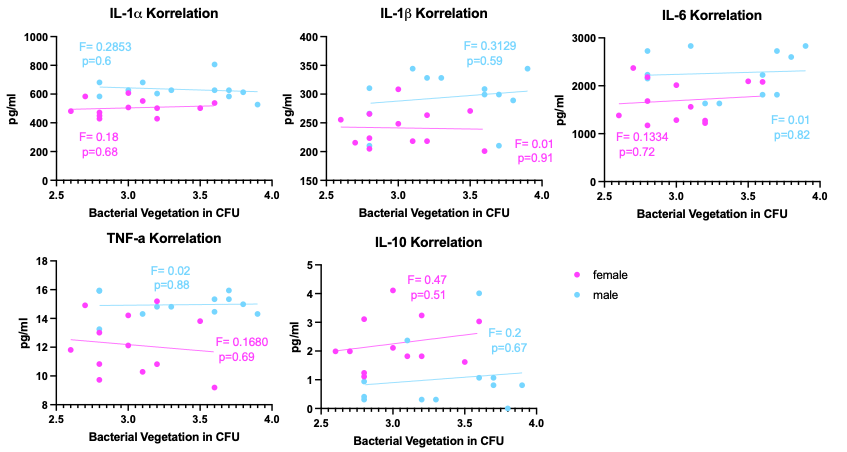


**S1 Correlation analyses between bacterial load and systemic inflammation** Correlation analyses was performed between plasma cytokine levels and valvular bacterial burden 24h after bacterial challenge
